# Reestablishment of Active Immunity against HBV Graft Reinfection after Liver Transplantation for HBV-Related End Stage Liver Disease

**DOI:** 10.1155/2014/764234

**Published:** 2014-12-17

**Authors:** Shi-Chun Lu, Tao Jiang, Wei Lai, Yuan Liu, Jing Zhang, Dao-Bing Zeng, Chuan-Yun Li, Meng-Long Wang, Dong-Dong Lin, Yue Zhu, You-Ping Li, Ning Li

**Affiliations:** ^1^Department of Hepatobiliary Surgery and Liver Transplantation Program, Beijing You-An Hospital, Capital Medical University, Beijing 100069, China; ^2^Institute & Hospital of Hepatobiliary Surgery, Key Laboratory of Digital Hepatobiliary Surgery of Chinese PLA, Chinese PLA Medical School, Chinese PLA General Hospital, 28 Fuxing Road, Beijing 100853, China; ^3^Department of General Surgery, Chengdu First People's Hospital, Sichuan 610041, China; ^4^Key Laboratory of Transplant Engineering and Immunology of Health Ministry, West China Hospital, Sichuan University, Chengdu, Sichuan 610044, China

## Abstract

*Background*. The aim of this study was to establish a hepatitis B virus (HBV) vaccination protocol among orthotopic liver transplantation (OLT) recipients under the coverage of a low-dose hepatitis B immunoglobulin (HBIG) combined with an antiviral agent prophylaxis protocol.* Method*. Two hundred OLT recipients were included in this study. The vaccine was injected at months 0, 1, 2, and 6. Low-dose HBIG combined with antiviral agent prophylaxis protocol was continued before reestablishment of active immunity against HBV in order to maintain a steady anti-HBs titer.* Results*. Active immunity against HBV was reestablished in 50 patients, for an overall response rate of 25%. Of the 50 patients, 24 discontinued HBIG without any HBV graft reinfection during a follow-up period of 26.13 ± 7.05 months. 21 patients discontinued both HBIG and antiviral agents during a follow-up period of 39.86 ± 15.47 months, and 4 patients among them appeared to be HBsAg positive. There was no recipient death or graft loss because of HBV reinfection.* Conclusions*. Vaccination preventing HBV reinfection for OLT recipients is feasible. The strategy withdrawal of HBIG with induction of active immunity against hepatitis B is reasonable for long-term survivors of OLT; however, discontinuation nucleoside analogues should be cautious.

## 1. Introduction

Hepatitis B virus- (HBV-) related end stage liver diseases (ESLD) account for over 80% of orthotopic liver transplantations (OLTs) in China, and active HBV replicative status prior to OLT is present in over 50% of patients. Although HBV graft reinfection and hepatitis B (HB) recurrence have been controlled to an acceptable level after the adoption of antiviral drugs such as the nucleoside analogue lamivudine (LAM) combined with hepatitis B immunoglobulin (HBIG) [1–3], drug resistance requiring the lifelong use of antiviral agents and virus escape under long-term use of HBIG may increase the risk of graft reinfection and HB recurrence [3–6]. Additionally, lifelong use of these drugs is associated with significant financial burden and inconvenience. Thus, a more rational, economical, and effective prevention regimen is needed.

Induction of active immunity against HBV has become a potential alternative in posttransplant patients who have undergone OLT for HBV-related liver diseases. To date, a few pilot studies have confirmed the feasibility of this method [7–11]. However, its effectiveness and response rate require further improvement [12–15]. Based on basic and clinical research of the induction of active immunity against HBV after OLT during the past decade, we have conducted this prospective clinical study regarding active immunization against hepatitis B virus graft reinfection in our center.

## 2. Patients and Methods

### 2.1. Study Design

This study was a prospective clinical study and was approved by the Ethics Committee of Beijing You-An Hospital (on January 4, 2006) and was performed according to the ethical guidelines of the 1975 Declaration of Helsinki. Study subjects were posttransplant patients who underwent OLT for HBV-related ESLD from 1999 to 2010. The written signed informed consent was obtained from all donors and recipients before surgery. Living and deceased donations were voluntary and altruistic in all cases. All organ donations or transplants were approved by the Institutional Review Board of Beijing You-An Hospital, Capital Medical University, under the guidelines of the Ethics Committee of the Hospital, the current regulations of the Chinese Government, and the Declaration of Helsinki.

**Figure 1:**
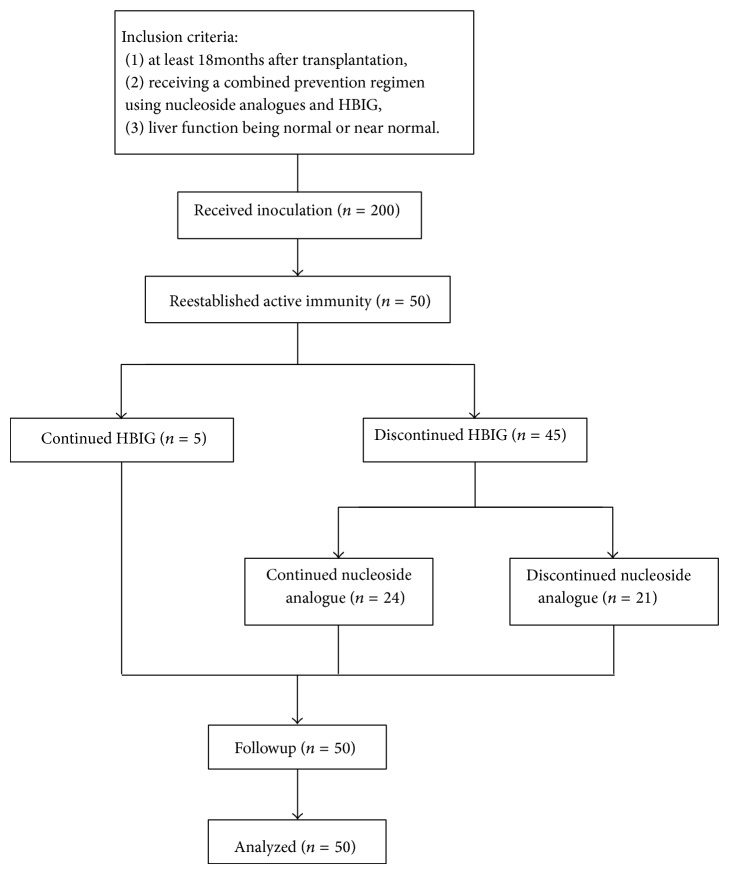
The study flowchart.

All patients were registered and followed up in Beijing You-An Hospital. Specific inclusion criteria were as follows: (1) at least 18 months following transplantation; (2) receiving a combined prevention regimen using nucleoside analogues and HBIG; and (3) liver function being normal or near normal. The study was clearly explained to all participants, and they all provided written signed informed consent. The two vaccines, Twinrix and Engerix-B, are officially approved for clinical use and are commercially available worldwide and in China. The study flowchart was shown in Figure 1.

The participant recruitment period was from January 1, 2005, to January 1, 2012. The clinical trial was started from February 1, 2006. And follow-up period ranges from June 10, 2006 (from the first successful vaccination), to December 31, 2013. The registration number in http://www.chictr.org/cn/ is CHiCTR-PNC-10001706.

### 2.2. Vaccine and Vaccination Schedule

The vaccines used were a recombinant hepatitis B surface antigen (HBsAg) vaccine containing HBsAg 20 *μ*g per vial (Engerix-B; GSK) and a bivalent vaccine which contained inactivated hepatitis A virus antigen and recombinant HBsAg 20 *μ*g per vial (Twinrix, GSK). One round of inoculation consisted of intramuscular injections of the vaccine (20 *μ*g for each inoculation) in the region of the triceps muscle at 0, 1, 2, and 6 months. All participants received at least one round of inoculation and were given multiple rounds of inoculation according to response status and anti-HBs level. The interval between two rounds of inoculation was 3 months.

### 2.3. Virological Assays

Serum HBV markers including anti-HBs were detected quantitatively with an electrochemiluminescence immunoassay (Roche Diagnostics GmbH, Mannheim, Germany) using a Cobas E 601 (Roche Diagnostics GmbH, Mannheim, Germany) immunoassay analyzer. HBV DNA was detected with a real-time quantitative PCR diagnostic kit for quantification of hepatitis B virus DNA (Shanghai Kehua Bio-Engineering Co., Ltd., China) using an ABI 7500 PCR instrument (Applied Biosystems, USA). The detection limit was 10^3^–10^7^ copies/mL.

### 2.4. Baseline Anti-HBs Titer

The combined regimen using nucleoside analogues and HBIG was maintained during the inoculation period. Participants received intramuscular injections of HBIG (400 IU/injection) regularly in order to maintain a stable baseline level of HBIG. Each vaccine inoculation was carried out 14 days after scheduled HBIG, and serum anti-HBs concentrations were measured on the day prior to the inoculation.

### 2.5. Definition of Responder

The patient was defined as a responder if the sera anti-HBs titer increased more than 100% above the baseline value during any vaccination course lasting more than 3 months or the elevated serum HBsAb titer remained high although it is less than 100% of baseline level. As soon as a patient was defined as a responder, exogenous HBIG administration was discontinued.

### 2.6. Withdrawal of HBIG and/or Nucleoside Analogue Protocol

Exogenous HBIG was withdrawn in patients with successful vaccination as described above. Another 3-month observation after exogenous HBIG was withdrawn, nucleoside analogues were then withdrawn subsequently if the patients were willing to stop nucleoside analogues. To maintain sustainable and spontaneous high anti-HBs production after discontinuation of HBIG, a booster vaccination was administered (usually every 3 to 6 months) in the early phase for successful responders. Regular surveillance of sera HBV DNA and HBV antigens was performed every 3 months. If it was found that the HBsAg turned to be positive during the follow-up period, nucleoside analogues were reused and/or intravenous injection of HBIG (2000 IU) was performed.

### 2.7. Statistical Analysis

Quantitative data were shown as mean ± standard deviation. Intergroup variations were analyzed with *t*-test or analysis of variance (ANOVA), and the percentage variation was analyzed with *χ* ^2^ test. SPSS 17.0 (SPSS Inc., Chicago, IL, USA) was used for all statistical analyses. The significance level *α* was set at 0.05.

## 3. Results

### 3.1. Clinical Data and Response to Vaccination

Until December 31, 2013, two hundred OLT recipients who met the inclusion criteria were enrolled in this study, and all received at least one round of inoculation. Fifty patients, including 41 males (82%) and 9 females (18%), achieved successful reestablishment of active immunity against HBV and were classified as responders. Among the 50 recipients, with a mean postoperative time of 35.8 ± 19.12 months (median: 27 months; range: 12 to 85 months), 19 cases were HBV active replicative (sera HBV DNA ≥ 10^3^ copies/mL) and 31 cases were HBV nonactive replicative (sera HBV DNA < 10^3^ copies/mL) before OLT.

Among the 50 successful cases, 20 received the Engerix-B vaccine and 30 received the Twinrix vaccine. The baseline anti-HBs titer of the 50 patients was 87.71 ± 38.82 IU/L (median: 83.61 IU/L; range: 23.90 to 195.30 IU/L). The mean anti-HBs titer was 264.91 ± 197.66 IU/L (median: 198.64 IU/L; range: 43.45 to 1000 IU/L) at the time when they were classified as responders. The average number of doses administered at the time of establishment of immunity was 5.06 ± 2.39 (median: 5; range: 1 to 11), with 22 cases requiring one round of inoculations, 22 requiring two rounds, and six requiring three rounds.

The highest anti-HBs titer in the follow-up period was 488.07 ± 322.52 IU/L, which was higher than that at the time of successful response (264.91 ± 197.66 IU/L, *t* = 4.172, and *P* = 0.000). The lowest anti-HBs titer in the follow-up period was 111.82 ± 74.53 IU/L, which was higher than the baseline anti-HBs titer (87.71 ± 38.82 IU/L, *t* = −1.965, and  *P* = 0.053). Booster vaccinations were necessary in some cases. The mean number of booster vaccinations administered was 1.61 ± 0.79 (median: 1; range: 1 to 4), and the anti-HBs titer increased to 438.09 ± 296.96 IU/L in these patients, which was similar to the highest sera anti-HBs titer (488.07 ± 322.52 IU/L, *t* = 0.751, and *P* = 0.455) in the follow-up period. The related data are shown in Tables 1 and 2.

### 3.2. Withdrawal of HBIG and/or Nucleoside Analogues

The interval of time between successful establishment of immunity and withdrawal of HBIG was 3.53 ± 4.00 months (median: 2 months; range: 1 to 22 months), and the interval of time between withdrawal of HBIG and withdrawal of both HBIG and antiviral agent was 5.57 ± 3.93 months (median: 3 months; range: 3 to 17 months). When HBIG was withdrawn, the mean anti-HBs titer was 257.72 ± 160.22 IU/L (median: 194.50 IU/L; range: 59.65 to 800 IU/L), which was higher than the mean baseline anti-HBs titer of 87.71 ± 38.82 IU/L (median: 83.61 IU/L; range: 23.90 to 195.30 IU/L) (*t* = −7.273, *P* = 0.000) but lower than the highest mean anti-HBs titer of mean 488.07 ± 322.52 IU/L (median: 388.15 IU/L; range: 95.81 to 1000 IU/L) (*t* = 4.333, *P* = 0.000).

There was no HBV graft reinfection or HB recurrence in the 24 cases who discontinued HBIG during the follow-up period of 26.13 ± 7.05 months (median: 24.5 months; range: 19 to 52 months), and 21 cases discontinued both HBIG and nucleoside analogues during the follow-up period of 39.86 ± 15.47 months (median: 34 months; range: 20 to 87 months). Five patients did not agree to discontinue HBIG and/or antiviral agents.

The mean anti-HBs titer of the 45 cases at the end of the followup was 341.36 ± 262.56 IU/L (median: 286.55 IU/L; range: 11.84 to 1000 IU/L), which was higher than that when HBIG was withdrawn (*t* = −1.829, *P* = 0.071), although the difference was not significant.

### 3.3. Comparison of the Engerix-B and Twinrix Groups

**Figure 2:**
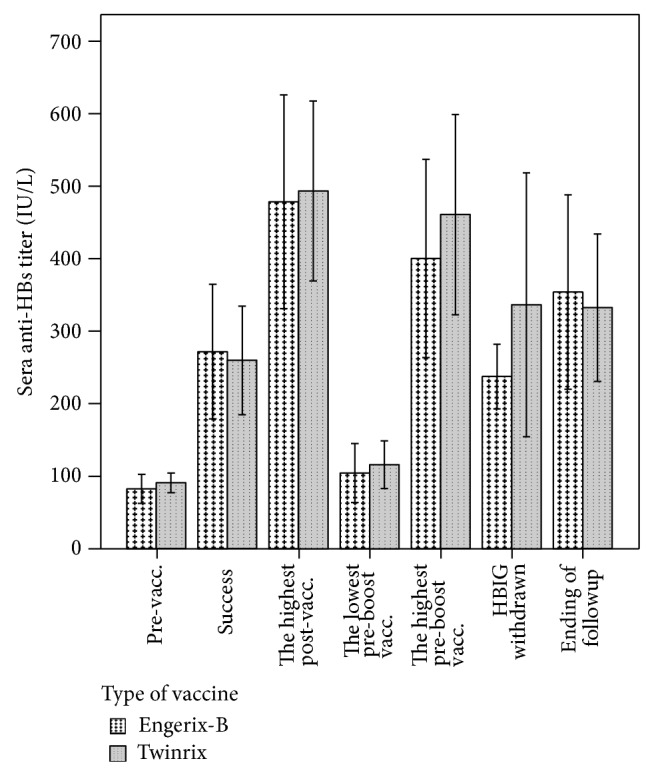
No significant differences were present in baseline anti-HBs titer, titer at successful establishment of immunity, the highest titer after vaccination, the lowest titer before booster vaccination, the highest titer after booster vaccination, titer when HBIG was withdrawn, and the titer at the end of followup (vacc.: vaccination).

There were 20 cases in Engerix-B group and 30 cases in Twinrix group in which active immunity against HBV was established. There was no difference between the two groups in baseline titers, titer at success of immunization, the highest titer, the lowest titer before booster vaccination, the highest titer after booster vaccinations, titer when HBIG was withdrawn, and the titer at the end of followup (Figure 2). The number of inoculation cycles required for success (1.75 ± 0.64 versus 1.63 ± 0.72, *t* = 0.587, and *P* = 0.560), dosage number (4.95 ± 2.14 versus 5.13 ± 2.57, *t* = −0.264, and *P* = 0.793), and number of booster vaccinations (1.86 ± 1.03 versus 1.46 ± 0.59, *t* = 1.528, and *P* = 0.135) were similar between the two groups. The number of cases requiring booster vaccinations (15 cases in the Engerix-B group and 24 cases in the Twinrix group) was also similar (*χ* ^2^ = 0.175, *P* = 0.467). However, the drug withdrawal rate of the Twinrix group was greater than that of Engerix-B group (*χ* ^2^ = 13.923, *t* = 0.001; Table 3).

### 3.4. Influences of Primary Disease on Vaccination Response

**Figure 3:**
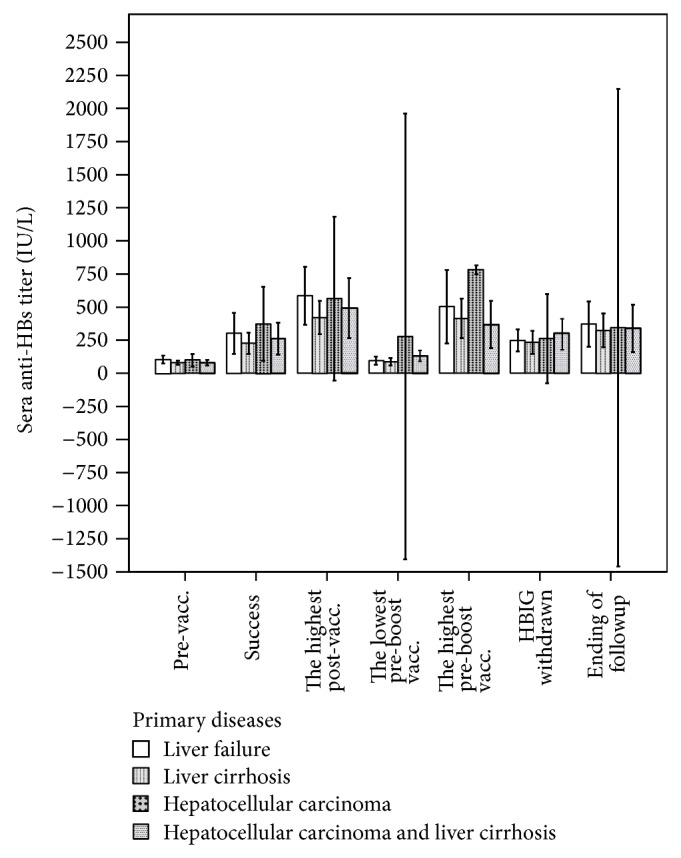
There were no significant differences among patients with LF, HCC, LC, and HCC combined with LC in baseline anti-HBs titer, titer at successful establishment of immunity, the highest titer after vaccination, the highest titer after booster vaccination, titer when HBIG was withdrawn, and titer at the ending of followup. The lowest titer of the HCC group before booster vaccination was higher than that of the other groups (94.81 ± 3.84 IU/L versus 87.40 ± 57.29 IU/L, 277.90 ± 187.38 IU/L, 131.74 ± 59.90 IU/L, *F* = 6.110, and *P* = 0.002, vacc.: vaccination).

In the 50 patients that had a successful response, there were no differences in those who had liver failure, hepatocellular carcinoma (HCC), liver cirrhosis (LC), and HCC combined with LC in success rate, titer at successful response, the highest titer, the lowest titer before booster vaccination, the highest titer after booster vaccination, titer when HBIG was withdrawn, and the titer at the end of followup (Figure 3).

### 3.5. Influences of Donor Anti-HBs Status on Vaccination Response

**Figure 4:**
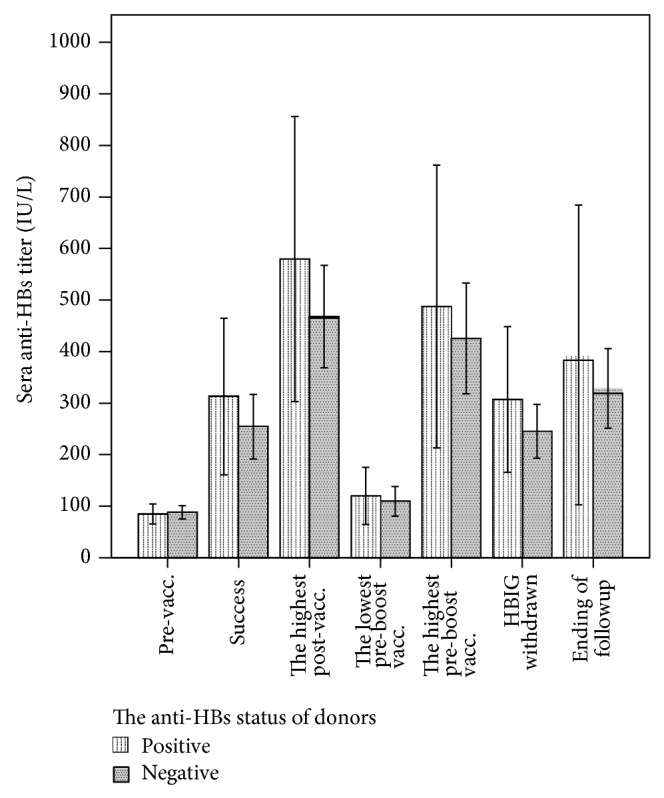
There were no significant differences between the anti-HBs positive and negative donor groups in titer at successful establishment of immunity, the highest titer after vaccination, the lowest titer before booster vaccination, the highest titer after booster vaccination, titer when HBIG was withdrawn, and the titer at the end of followup (vacc.: vaccination).

The influence of the donor anti-HBs status (positive or negative) on the response of the recipients to vaccination is shown in Figure 4. There were no differences between the positive and negative groups in titer at successful response, the highest titer, the lowest titer before booster vaccination, the highest titer after booster vaccination, titer when HBIG was withdrawn, and titer at the end of followup. In addition, the round of inoculation when successful immunization occurred, the number of doses, the number of booster vaccinations, and the booster vaccination rate of the two groups were similar (data not shown).

### 3.6. Influences of Recipient Preoperative HBV DNA Titer on Vaccination Response

**Figure 5:**
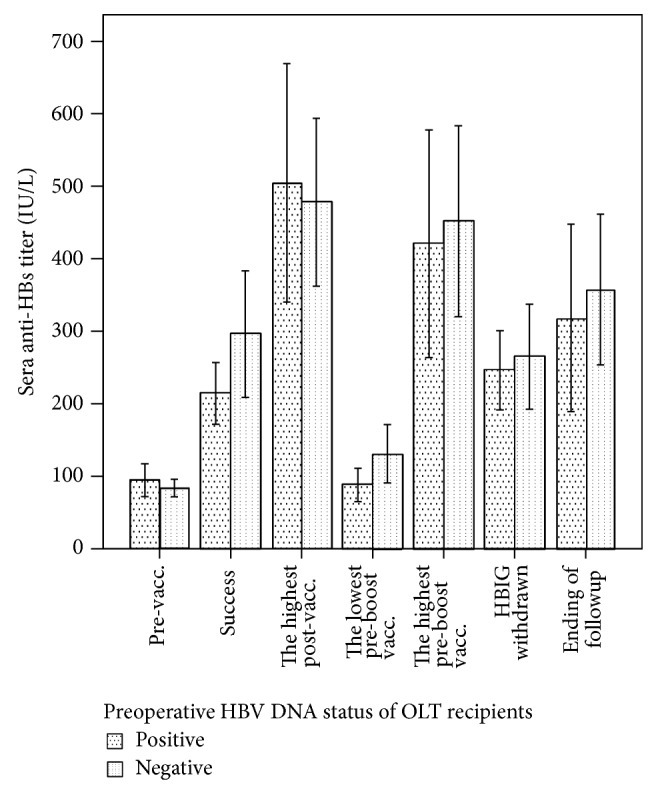
There were no significant differences between the ≥10^3^ copies/mL group and the <10^3^ copies/mL group in baseline anti-HBs titer, titer at successful establishment of immunity, the highest titer after vaccination, the lowest titer before booster vaccination, the highest titer after boost vaccination, titer when HBIG was withdrawn, and the titer at the end of followup (vacc.: vaccination).

Recipients' preoperative sera HBV DNA level (≥10^3^ copies/mL or <10^3^ copies/mL) did not influence the response to vaccination. There were no differences between the ≥10^3^ copies/mL group and <10^3^ copies/mL group in baseline titer, titer at successful immunization, the highest titer, the lowest titer before booster vaccination, the highest titer after booster vaccination, titer when HBIG was withdrawn, and the titer at the end of followup (Figure 5). In addition, the round of inoculation when successful immunization occurred, the number of doses, the number of booster vaccinations, and the booster vaccination rate of the two groups were similar (data not shown).

### 3.7. HBV Reinfection in Patients Withdrawn from HBIG and Nucleoside Analogues Regimen

In the 21 patients who discontinued HBIG and nucleoside analogues, 4 patients appeared HBsAg positive. The interval of time between withdrawal of nucleoside analogues and hepatitis B virus reinfection was 14.25 months (6 to 23 months). The four patients reused nucleoside analogues and one patient whose HBsAg and HBV DNA were negative was treated with HBIG (2000 IU). There was no recipient death or graft loss because of HBV reinfection. The gene mutations of HBV were detected by nested PCR assay and gene sequence analysis and the clinical characteristics of these 4 patients were reviewed (Table 4). Additionally, we notified other patients withdrawn from HBIG and nucleoside analogues regimen that they had the risk of HBV reinfection, although the risk was unclear. They could reuse nucleoside analogues according to their wishes.

## 4. Discussion

Whether HBIG and nucleoside analogues administered for the prevention of HBV graft reinfection and HB recurrence after OLT for HBV-related ESLD can be withdrawn or not has yet to be determined. Based on the latest findings [16] and the results from our previous studies [17–19], the answer is no; they cannot be withdrawn. Long-term or lifelong use of these drugs raises a series of issues, including resistance to antiviral drugs, HBIG-induced HBV immune escape, and high monetary costs. Thus, an alternative protocol which is more rational, economical, and effective is needed.

Since active immunization is the gold standard for preventing HBV infection among the general population, induction of active immunity against HBV after OLT appeared to be an effective alternative to HBIG and nucleoside analogues. While there are multiple clinical reports on the induction of active immunity against HBV in patients undergoing OLT for HBV-related ESLD [7–15], most studies only included a small number of cases, and all were nonactive HBV replicative. In addition, participants stopped or did not use exogenous HBIG in order to facilitate a response to the vaccine [7, 8, 11–13]. Our study included 200 patients who had completed at least one vaccination round, and our results showed that establishment of active immunity against HBV after OLT caused by HBV-related ESLD is feasible, especially including recipients with HBV active replication before surgery.

According to literature reports, adoptive immunity is helpful for the reestablishment of active immunity against HBV after OLT. The primed lymphocytes from HBsAb-positive donor's liver graft are beneficial to the induction of positive immunity against HBV. Memory T lymphocytes and memory B lymphocytes play the most important role in this process; they are activated by HBV antigens such as HBsAg in the donors. After OLT, they are stimulated by the HBV vaccine in the recipients and lymphocyte proliferation occurs, which consequently leads to the active immunity against HBV. Unfortunately, reports showed that the adoptive immunity is generally ineffective, and the response intensity to HBV vaccination is relatively weak and short-term [13]. Similarly, we did not find significant adoptive immunity in the present study as there was no significant difference when comparing HBsAb-positive donors and HBsAb-negative donors.

We also observed that the response intensity of the HCC group was greater than that of the other disease groups, especially after booster vaccinations were performed, though the differences were not significant (Figure 2). To prevent HCC recurrence, immunosuppressive agents such as tacrolimus were reduced to the possible lowest dosage, which was the reason why the response intensity was greater for the HCC group than other groups. This may be an advantage for establishing active immunity against HBV after OLT.

Previous study has indicated that the HBV vaccine type could affect the final immune response outcome. The vaccination efficiency of recombinant HBsAg is only 7.7% [13], but vaccination efficiency is increased to 47% when the Sci-B-Vac complex vaccine containing pre-S antigen is used [20]. The Binzle adjuvant vaccine also effectively improves the vaccination response rate [10]. These results indicate that improvements in vaccines can enhance the immune response and suggest that a combination vaccine has the potential to improve the vaccination response rate [21]. However, the adjuvant vaccine mentioned above is still not commercially available, and its safety requires clarification. In our study, the Twinrix group exhibited a greater response intensity than the Engerix-B group, and the drug withdrawal rate of the Twinrix group was greater than that of the Engerix-B group. The reason for these differences may be that Twinrix is a bivalent vaccine which contains inactivated hepatitis A virus antigen and recombinant HBsAg, while Engerix-B is a monovalent vaccine which only contains recombinant HBsAg. This difference could result in a bystander effect caused by hepatitis A virus antigen, which facilitates the immune response for HBV vaccination. In addition, our patients did not stop HBIG during inoculation. It is possible that the presence of the HBsAg-HBIG complex further enhanced the effectiveness of the vaccines [22].

In this study, most patients with successful vaccination had a successful reaction after 5 to 11 doses. This is because all of the transplant recipients were immunocompromised, whose immune systems had different levels of native/innate tolerance or acquired tolerance to HBsAg. Their immune responses could only be restored through long-term and continuous antigenic stimulation. This is another example illustrating that the immune response can be improved along with the improvement of general conditions after liver transplantation. This suggests that inoculations should be continued until successful vaccination is achieved among immunocompromised subjects. In this study, long-term and repetitive vaccine stimulation was an important method to create and cultivate an enhanced immune response in the immunocompromised individuals. Intermittent vaccination reinforcement (booster vaccination) was also an important means to maintain a spontaneous anti-HBs production.

The average length of time after transplantation was 35.80 ± 19.12 months in the patients with successful vaccinations in this study. Our previous study [21] confirmed that the general condition of OLT recipients and the antigen-presenting ability of the primary immune cells (dendritic cells) exhibited a tendency towards improvement with elongation of the postoperative time and minimization of the immunosuppression, which was essentially the immunological basis of this phenomenon. Therefore, from an immunological perspective the longer the postoperative time and the better the recovery of immune function, the higher the success rate of vaccination. However, the risk of viral resistance to drugs and immune escape are higher with the elongation of the postoperative prevention regimen. We believe that patients should be vaccinated before the occurrence of those aforementioned phenomena. We have named this time period the “opportunity window” to reconstruct active immunity. Therefore, timely vaccination is the wisest choice. Our results also suggest that it is reasonable to initiate reconstruction of active immunity 18 months after transplantation.

Similarly, the reaction intensity was different in patients with responses. As discussed above, responses were defined based on the reaction intensity. In immunocompromised individuals with responses, antibody production was characterized by low intensity, short duration, and the need of regular vaccination reinforcement. We observed that, during the process of HBIG and antiviral drug withdrawal, it was necessary to give one reinforcing inoculation regularly in the early stage in order to maintain a sustainable and spontaneous anti-HBs production over baseline level, which was especially important after entering the withdrawal protocol.

The reasons for HBV reinfection in patients who withdrew HBIG and nucleoside analogues were complex. First, it was shown that mutation for HBsAg escape under immune pressure or drug resistance mutations have occurred before liver transplantation. Second, traces of hepatitis B virus still existed in liver, myeloid element, or other tissues. Third, the antibodies, by which the patients reestablished active immunity against HBV generated, could not cover all subtypes of hepatitis B virus. We will evaluate the status of immunization and hepatitis B virus before and after vaccination in future research.

In conclusion, induction of active immunity against HBV in patients who have undergone OLT for HBV-related ESLD is feasible. For long-term posttransplant survivors, withdrawal of HBIG with induction of active immunity against hepatitis B is reasonable, effective, dependable, and economical; however, discontinuation nucleoside analogues should be cautious. But because of the complexity of the immune response and the molecules involved in HBV immunology further study is needed; moreover, the indication for withdrawal of nucleoside analogues needs further exploration.

## Figures and Tables

**Table 1:** Demographic, etiological, and virological features of the 50 cases with a successful response before OLT.

Age (y)	50.82 ± 8.58 (52.00, 27–69)
Gender (M/F)	41/9
Postoperative time (months)	35.80 ± 19.12 (27.00, 12.00–85.00)
Diagnosis	
Liver failure (*n*, %)	12, 24%
Liver cirrhosis (*n*, %)	22, 44%
HCC (*n*, %)	4, 8%
Liver cirrhosis and HCC (*n*, %)	12, 24%
HBsAg (+)/(−)	50/0
Sera HBV DNA preoperation ≥10^3^/<10^3^ copies/mL	19/31

**Table 2:** Data of the 50 cases at the time active immunity against HBV was reestablished.

Vaccine (Engerix-B/Twinrix)	20/30
Baseline anti-HBs titers (IU/L)	87.71 ± 38.82 (83.61, 23.90–195.30)
Number of cycles for reestablishment of immunity (*n*, %)	First cycle: 22, 44%
	Second cycle: 22, 44%
	Third cycle: 6, 12%
Number of doses for reestablishment of immunity	5.06 ± 2.39 (5.00, 1.00–11.00)
Anti-HBs titer at reestablishment of immunity (IU/L)	264.91 ± 197.66 (198.64, 43.45–1000.00^*^)
The highest anti-HBs titer (IU/L)	488.07 ± 322.52 (388.15, 95.81–1000.00^*^)
The lowest anti-HBs titer^#^ (IU/L)	111.82 ± 74.53 (93.34, 25.53–410.40)
Number of booster vaccinations^#^	1.61 ± 0.79 (1.00, 1–4)
The highest titer of sera anti-HBs after booster vaccination (IU/L)	438.09 ± 296.96 (313.70, 75.84–1000.00)

^*^The upper limit of sera anti-HBs quantitative detection by ELISA (Roche) is 1000 IU/L.

^#^The anti-HBs titer of 39 recipients had dropped to <100 IU/L or declined by 50% during the follow-up period, so the booster vaccinations were performed.

**Table 3:** Drugs withdrawn according to different factors.

		Drugs withdrawn		
		None	HBIG	HBIG and nucleoside analogues
Donor anti-HBs status	Positive	0	6	3
	Negative	5	18	18

Vaccine type	Engerix-B	4	3	13
	Twinrix	1	21	8

Recipient HBV DNA before OLT (copies/mL)	≥10^3^	2	7	10
	<10^3^	3	17	11

Primary disease before OLT	Liver failure (LF)	0	7	5
	Liver cirrhosis (LC)	4	9	9
	Hepatocellular carcinoma (HCC)	1	2	1
	HCC and LC	0	6	6

**(a):** 

Case	Subtype of HBV	Mutation in the region of HBV polymers	Mutation in the region of HBV gene S	Mutation in the region of HBV gene PreS/S	BCP mutation			HBV DNA before OLT (copies/mL)	Antiviral agent before OLT
					A1762T	G1764A	G1896A		
1	C1	—	P120PQ, M133MT, G145R	—	Negative	Negative	Negative	negative	LAM
2	C2	—	—	—	Negative	Negative	Negative	1.00*E* + 04	LAM
3	B2	N236T	Q129R	PreS2 TGTACTTTC (46–54 nt) deletion	Positive	Positive	Negative	negative	ETV
4	B2	—	G145R	—	Negative	Negative	Negative	2.97*E* + 09	ADV

**(b):** 

Case	Vaccine type	Number of doses for reestablishment of immunity	Antiviral agent after OLT	Interval of time between withdrawal of nucleoside analogues and HBV reinfection (month)	HBV DNA at the time of HBV reinfection (copies/mL)	Current antiviral agent	Current HbsAg	Current HBV DNA (copies/mL)
1	Twinrix	5	ADV	12	5.23*E* + 06	TDF	Positive	Negative
2	Twinrix	6	ADV	6	1.00*E* + 06	ETV	Positive	Negative
3	Twinrix	10	ETV	16	<2.00*E* + 1	ETV	Negative	Negative
4	Engerix-B	6	ADV	23	2.92*E* + 03	TDF	Positive	7.60*E* + 01
